# Clinical Utility of 18F-FDG PET/CT for Staging and Restaging of Pediatric Malignant Melanoma: A Single-Center Retrospective Study

**DOI:** 10.3390/children13101428

**Published:** 2026-10-18

**Authors:** Emine Goknur Isik, Rejin Kebudi

**Affiliations:** 1 Department of Nuclear Medicine, Istanbul Faculty of Medicine, Istanbul University, 34093 Istanbul, Türkiye; 2 Department of Pediatric Hematology and Oncology, Oncology Institute, Istanbul University, 34093 Istanbul, Türkiye

**Keywords:** pediatric malignant melanoma, childhood melanoma, 18F-FDG PET/CT, staging, restaging

## Abstract

**Highlights:**

**What are the main findings?**

- 18F-FDG PET/CT demonstrated high specificity (95.2%) and negative predictive value (90.9%) for detecting clinically active disease in pediatric malignant melanoma.
- Spitzoid melanoma, representing 25% of the cohort, showed predominantly negative PET/CT findings at initial staging, which may reflect relatively lower 18F-FDG avidity in this histological subtype.

**What are the implications of the main findings?**

- Negative 18F-FDG PET/CT findings may support clinical decision-making during restaging in pediatric melanoma; however, false-negative results occurred, and negative findings should be interpreted together with continued clinical follow-up.
- Whole-body 18F-FDG PET/CT provides complementary value in pediatric melanoma beyond primary tumor detection, enabling comprehensive assessment for occult nodal and distant metastatic disease.

**Abstract:**

**Background/Objectives:** Malignant melanoma is a rare entity in the pediatric population, accounting for approximately 1–3% of all childhood malignancies. Despite its rarity, it is the most common skin cancer in children. Accurate staging and monitoring are essential for treatment planning and prognosis. This study aimed to evaluate the clinical value of 18F-FDG PET/CT in staging and restaging of pediatric malignant melanoma. **Methods:** We retrospectively analyzed pediatric patients aged 18 years or younger who underwent 18F-FDG PET/CT imaging between 2010 and 2025 with histopathologically confirmed malignant melanoma. Demographic data, primary lesion localization, PET/CT findings, pathology reports, and clinical outcomes were recorded. Lesions were categorized as positive and negative in the final analysis. Diagnostic performance parameters (with 95% confidence intervals (CIs)) were calculated using histopathological confirmation or clinical follow-up as the reference standard, and categorical associations were assessed using Fisher’s exact test. **Results:** A total of 20 patients (12 females, 8 males; mean age: 11 years, range: 4–18) and 31 PET/CT examinations were included, with a mean follow-up duration of approximately 98 months. PET/CT was performed for initial staging in 17 examinations and for restaging in 14. On an examination-based analysis, PET/CT findings were negative in 22/31 (71.0%) and positive in 9/31 (29.0%) examinations. On a patient-based analysis, 12/20 (60%) patients had all examinations classified as true negative and remained disease-free throughout follow-up. 18F-FDG PET/CT demonstrated a sensitivity of 80.0% (95% CI: 49.0–94.3%), specificity of 95.2% (95% CI: 77.3–99.2%), positive predictive value of 88.9% (95% CI: 56.5–98.0%), negative predictive value of 90.9% (95% CI: 72.2–97.5%), and overall accuracy of 90.3% (95% CI: 75.1–96.7%) for the detection of clinically active disease. Agreement between PET/CT findings and clinical outcomes was substantial (Cohen’s κ = 0.77). Among the nine PET-negative restaging examinations, one was subsequently confirmed as false-negative; the remaining eight showed no evidence of active disease during the available follow-up period. **Conclusions:** In this exploratory retrospective study, 18F-FDG PET/CT demonstrated promising diagnostic performance for staging and restaging of pediatric malignant melanoma, with high specificity and negative predictive value. These findings should be interpreted with caution given the small sample size and retrospective design but suggest a potentially meaningful role for 18F-FDG PET/CT in the multidisciplinary management of this rare pediatric malignancy.

## 1. Introduction

Malignant melanoma is a highly aggressive form of skin cancer arising from melanocytes. Although it is extremely rare in the pediatric population, it accounts for 1–3% of all childhood malignancies and represents the most common skin cancer in children [1,2,3]. Pediatric melanoma differs from its adult counterpart in terms of biological behavior, clinical presentation, and prognosis [4]. Congenital melanocytic nevi and genetic predispositions have been recognized as potential risk factors in children [5,6].

Despite its rarity, pediatric melanoma can exhibit aggressive features, including regional lymph node involvement and distant metastasis [7,8]. Early and accurate staging is essential to determine prognosis and guide therapy. Conventional imaging techniques may be limited in detecting subclinical metastases, particularly in early recurrence.

Sentinel lymph node biopsy constitutes the cornerstone of regional nodal staging in melanoma. However, imaging plays a critical role in evaluating systemic disease, particularly in patients with advanced-stage disease, suspected recurrence, or during follow-up after treatment [9]. In pediatric melanoma, where evidence-based imaging guidelines are limited and management strategies are frequently adapted from adult protocols [2,8], reliable whole-body imaging may have a meaningful impact on clinical decision-making.

18F-fluorodeoxyglucose positron emission tomography/computed tomography (18F-FDG PET/CT) is a powerful hybrid imaging modality combining metabolic and anatomical information. In adult patients with advanced cutaneous melanoma, 18F-FDG PET/CT has demonstrated high diagnostic performance for the detection of systemic metastases, with pooled sensitivity and specificity approaching 90%, as reported in a recent meta-analysis [10,11]. While 18F-FDG PET/CT has been extensively studied in adult melanoma for staging, restaging, and treatment response evaluation [12,13,14,15], data on its role in pediatric cases remain scarce due to the low incidence of the disease [2,3,7]. In this study, we aimed to evaluate the clinical utility of 18F-FDG PET/CT in children diagnosed with malignant melanoma.

## 2. Methods

### 2.1. Study Design and Patients

This was a retrospective study including pediatric patients aged 18 years or younger who underwent 18F-FDG PET/CT imaging between 2010 and 2025 at a single center. All patients had histopathologically confirmed malignant melanoma. According to age-based classification, patients aged 1–11 years were categorized as children and those aged 12–18 years as adolescents. The study was approved by the institutional ethics committee (2025/563).

### 2.2. Data Collection

**Figure 1:**
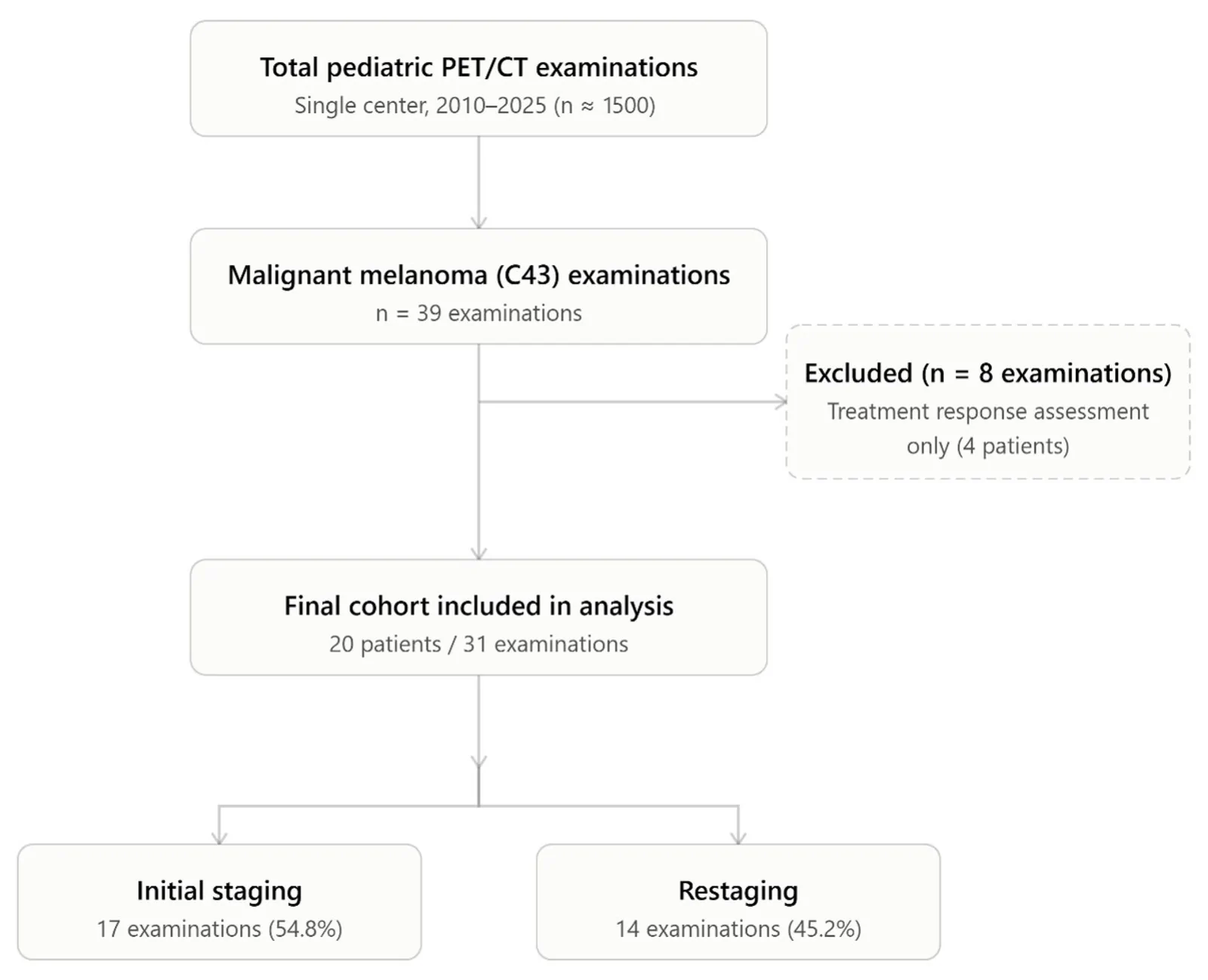
Study flow diagram.

Demographic characteristics (age, sex), lesion characteristics (localization, melanoma subtype), and the presence of congenital melanocytic nevi were recorded. Each 18F-FDG PET/CT scan was classified according to its clinical indication as one of the following: initial staging (S) or restaging (RS). A total of 31 PET/CT scans from 20 patients were included in the analysis. PET/CT examinations performed solely for treatment response assessment were not included in the final analysis (Figure 1).

### 2.3. PET/CT Acquisition Protocol

All patients fasted for at least six hours prior to imaging, and blood glucose levels were confirmed to be below 200 mg/dL. 18F-FDG was administered intravenously according to the European Association of Nuclear Medicine (EANM) pediatric dosage card, using weight-based dose calculation [16]. The mean administered activity was 8.3 ± 2.6 mCi (approximately 307 ± 96 MBq; range: 148–481 MBq). PET/CT imaging was performed on two scanner systems over the study period: examinations acquired before 2019 were performed on a Biograph TruePoint PET/CT system (Siemens Healthcare, Erlangen, Germany) with standard OSEM reconstruction, while examinations from 2018 onwards were performed on a Discovery IQ PET/CT system (GE Healthcare Milwaukee, WI, USA), reconstructed using a Bayesian penalized likelihood reconstruction algorithm (Q.Clear, β = 300). PET/CT scans were performed approximately 60 min after 18F-FDG administration. Whole-body acquisitions from vertex to feet were obtained using 2 min per bed position, with an average of 7–10 bed positions depending on patient height. Low-dose CT with automated tube current modulation (mAs) was used for attenuation correction and anatomical localization; tube voltage was set automatically by the system based on patient weight. CT dose-length product (DLP) and individual effective dose estimates were not systematically recorded in the retrospective dataset. No sedation was used in any patient. All acquired data were transferred to the Advantage Workstation (version 4.7, GE Healthcare Milwaukee, WI, USA) for visual evaluation. Reconstructed PET, CT, and fused images were evaluated by two experienced nuclear medicine physicians.

### 2.4. Image Analysis

Each PET/CT scan was initially evaluated for the presence of pathological 18F-FDG uptake and categorized as positive (focal uptake exceeding background activity in a pattern consistent with active malignant disease), suspicious (focal uptake suggestive but not definitively consistent with malignancy), or negative (no abnormal uptake beyond physiological distribution). Four examinations were initially interpreted as suspicious. For the adjudicated analysis, these examinations were resolved through histopathological confirmation or clinical and imaging follow-up and assigned to the positive or negative category so that no examination remained equivocal in the final dataset. Because this adjudication incorporated reference standard information and may therefore introduce incorporation bias, diagnostic performance was additionally calculated from the initial interpretations alone, treating suspicious examinations first as negative and then as positive (Supplementary Table S1). Final PET/CT classifications were compared with histopathology or clinical/imaging follow-up to determine true-positive (TP), false-positive (FP), true-negative (TN), and false-negative (FN) results. In cases where histopathological confirmation was not available, PET/CT findings were correlated with clinical and imaging follow-up conducted at 3–6-month intervals within the routine oncological surveillance protocol. PET-negative examinations with no evidence of disease progression during follow-up were classified as true negatives.

### 2.5. Statistical Analysis

Descriptive statistics were used to summarize patient characteristics, PET/CT indications, and imaging findings. All statistical analyses were performed using IBM SPSS Statistics software (version 30.0). Categorical variables were expressed as numbers and percentages. Diagnostic performance of 18F-FDG PET/CT was assessed on an examination-based level using histopathology or clinical and imaging follow-up as the composite reference standard; sensitivity, specificity, positive predictive value (PPV), negative predictive value (NPV), and overall accuracy were calculated with 95% confidence intervals (CIs) using the Wilson score method. Agreement between 18F-FDG PET/CT findings and clinical outcomes was assessed using Cohen’s kappa coefficient; kappa values were interpreted according to the scale proposed by Landis and Koch [17], where values of 0.61–0.80 indicate substantial agreement. Associations between categorical variables (e.g., tumor localization and sex or age group) were assessed using Fisher’s exact test, given the small expected cell counts. An exploratory subgroup analysis of diagnostic performance was conducted separately for initial staging and restaging examinations. No survival analysis or inferential statistical comparisons were performed due to the limited sample size and descriptive nature of the study. For patient-based descriptive analysis, patients were categorized according to their overall examination profile. Examination-based confidence intervals were calculated using the Wilson score method and should be interpreted with caution, as they do not account for within-patient correlation arising from repeated examinations.

## 3. Results

### Patient Characteristics

**Figure 2:**
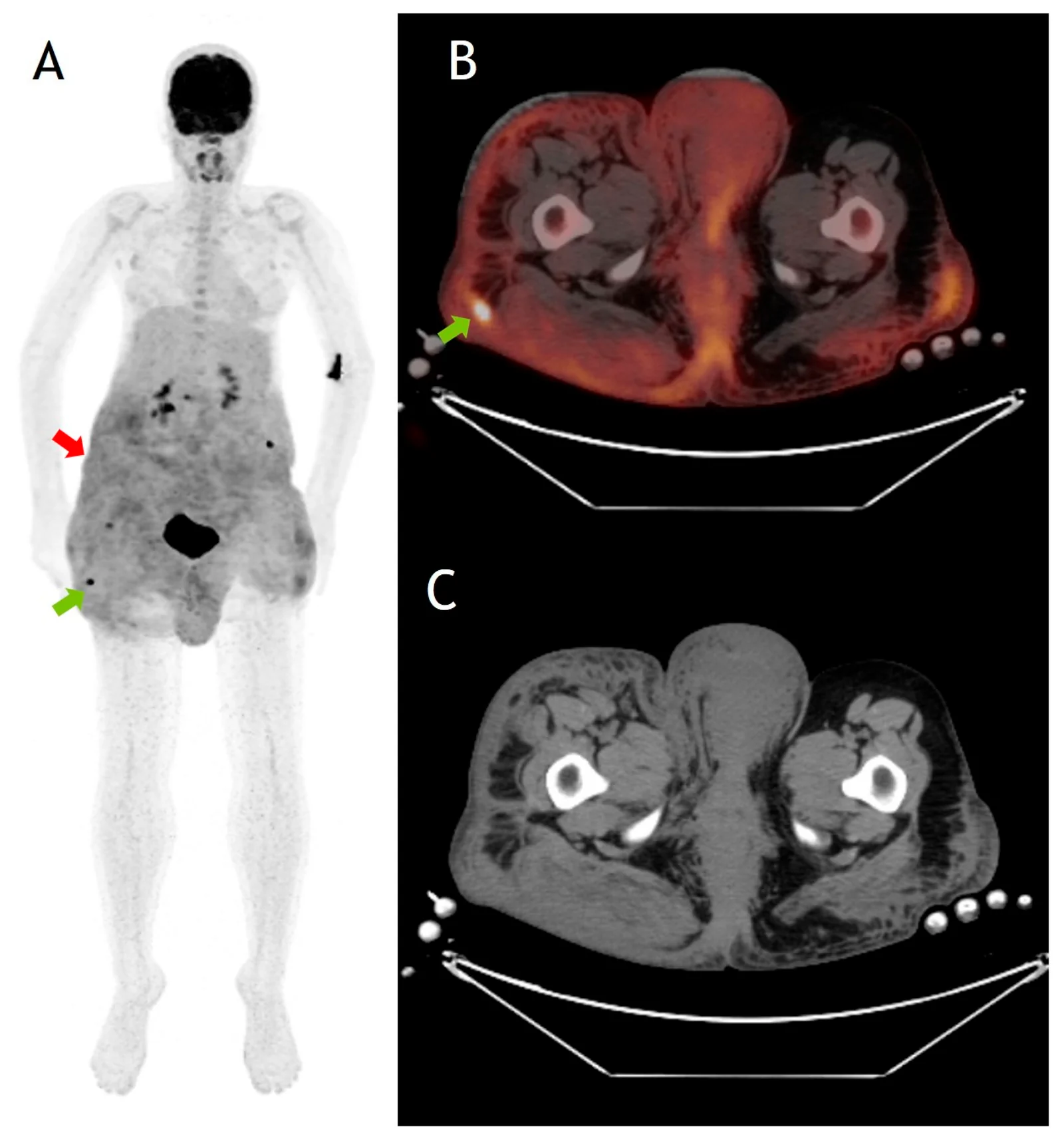
18F-FDG PET/CT findings in pediatric melanoma arising from a giant congenital melanocytic nevus. 18F-FDG PET/CT findings in a 16-year-old girl with cutaneous malignant melanoma arising in a giant congenital melanocytic nevus. Maximum intensity projection image (**A**) demonstrates a focal hypermetabolic lesion in the right gluteal region. Corresponding axial fused PET/CT (**B**) and CT (**C**) images confirm a soft-tissue mass with increased 18F-FDG uptake at this site, consistent with biopsy-proven melanoma. Red arrow: giant congenital melanocytic nevus. Green arrow: demonstrates a focal hypermetabolic lesion.

A total of 20 pediatric patients (12 females, 8 males) with malignant melanoma were included in the study. The mean age at imaging was 11 years (range: 4–18 years). In our cohort, 9 patients were classified as children and 11 as adolescents. Tumor localization did not differ significantly between male and female patients (Fisher’s exact test, *p* = 0.769) or between children and adolescents (*p* = 0.451). The mean follow-up duration was approximately 98 months (median follow-up after true-negative PET/CT examinations: 104 months; range: 16–186 months). Primary tumor locations included the lower extremities in 7 patients (35%), upper extremities in 5 (25%), trunk in 5 (25%), and head–neck region in 3 patients (15%). One patient had conjunctival melanoma, and five patients had melanoma arising in congenital melanocytic nevi (Figure 2), three located on the trunk and two on the extremities. Breslow thickness was reported in 11 patients (mean: 5.5 mm), and Clark level in 8 patients (mean: IV), reflecting the predominance of thick primary tumors in this cohort; complete histopathological reports were available for only 11 of 20 patients (55%), as the remainder were diagnosed at external institutions or during earlier years with less standardized reporting (Table 1). Melanoma subtypes included conventional melanoma (*n* = 9, 45%), spitzoid melanoma (*n* = 5, 25%), melanoma arising in a congenital melanocytic nevus (*n* = 5, 25%), and conjunctival melanoma (*n* = 1, 5%). Clinical stage distribution was as follows: Stage I in 6 (30%), Stage II in 5 (25%), and Stage III in 9 (45%) patients; no patient presented with Stage IV disease. Among true-positive examinations, the median Maximum Standardized Uptake Value (SUVmax) was 10.6 (range: 1.8–28.0).

A total of 31 18F-FDG PET/CT examinations were performed for initial staging and restaging purposes. Of these, 17 examinations (54.8%) were obtained for initial staging and 14 examinations (45.2%) for restaging due to clinical suspicion of recurrence.

**Figure 3:**
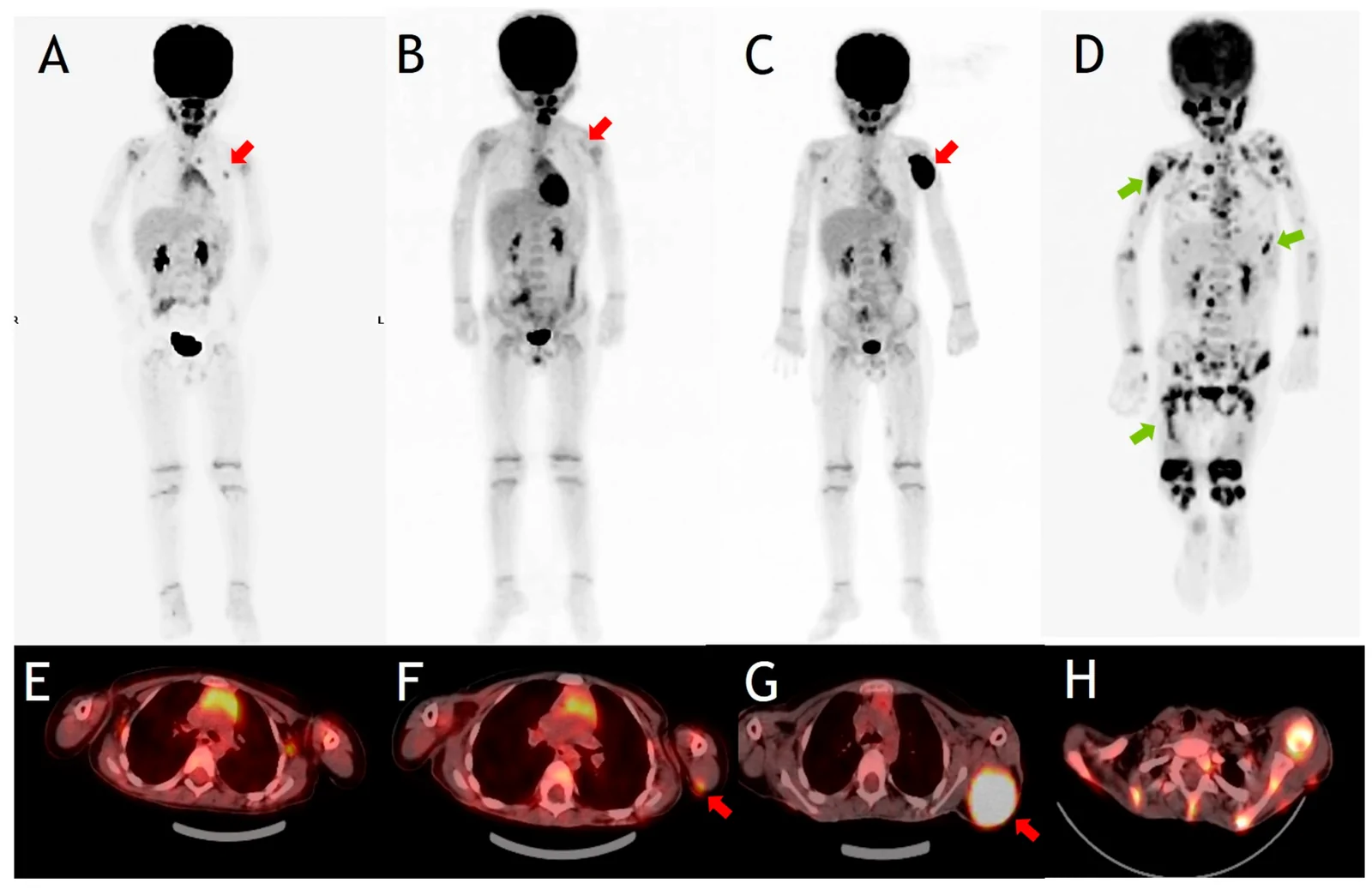
Serial 18F-FDG PET/CT images of a 4-year-old boy with malignant melanoma arising from a congenital melanocytic nevus of the trunk (Breslow thickness: 15 mm; Clark level V; Stage III), surgically excised at initial diagnosis. Maximum intensity projection images (**A**–**D**) and corresponding axial fused PET/CT images (**E**–**H**) demonstrate findings at initial staging ((**A**,**E**); red arrow, SUVmax 2.2), treatment response assessment ((**B**,**F**); red arrow, SUVmax 2.3), restaging ((**C**,**G**); red arrow, SUVmax 8.9), and subsequent disease progression ((**D**,**H**); red arrows, SUVmax 9.7), with eventual disease-related death. Green arrows in (**D**) indicate representative sites of widespread osseous metastases (SUVmax 9.5). Progressive increase in metabolic activity and development of disseminated metastatic disease are evident on serial imaging. Note that panels (**B**,**F**) correspond to a treatment-response assessment examination, which was excluded from the diagnostic performance analysis but is included here to illustrate the complete serial clinical course of this patient.

On a patient-based analysis, 12 of 20 patients (60%) had all examinations classified as true negative and remained disease-free throughout follow-up. In 6 patients (30%), at least one true-positive examination was recorded, indicating detection of active disease at some point during follow-up; one of these patients died of disease-related causes (Figure 3). False-negative results occurred in two patients (10%), and one patient (5%) had a single false-positive examination not confirmed on clinical follow-up. One patient had both a false-negative and a subsequent true-positive examination during follow-up. All eight true-positive examinations were confirmed by histopathological analysis of biopsy or surgical specimens, whereas the single false-positive examination was assessed by clinical and imaging follow-up. Among the six patients with at least one true-positive PET/CT examination, five underwent re-excision, two of whom also initiated systemic therapy following the detection of disease on PET/CT. Because management decisions were made in a multidisciplinary setting incorporating clinical and histopathological findings, the independent contribution of PET/CT to these decisions could not be determined. The single false-positive finding localized to a left cervical lymph node was not confirmed on clinical follow-up. In the 12 patients with consistently true-negative PET/CT examinations, the absence of disease was supported by clinical and imaging follow-up over a median of 104 months (range: 16–186 months), with no disease recurrence at the initially evaluated site.

On an examination-based analysis, PET/CT findings were interpreted as negative in 22 of 31 examinations (71.0%), positive in 9 examinations (29.0%), and no examination was classified as equivocal in the final analysis. Among the nine PET-positive examinations, local recurrence at the primary tumor site was identified in 1, isolated lymph node involvement in 5 (including 1 false-positive finding), combined local and nodal disease in 2, and distant metastatic disease in 1. The latter represented multiple lesions (bone, soft tissue, and lung) identified within a single examination in one patient (Table 2).

Using clinical assessment and follow-up as the composite reference standard, exploratory performance analysis on an examination-based level demonstrated a sensitivity of 80.0% (95% CI: 49.0–94.3%), a specificity of 95.2% (95% CI: 77.3–99.2%), a positive predictive value of 88.9% (95% CI: 56.5–98.0%), and a negative predictive value of 90.9% (95% CI: 72.2–97.5%) for the detection of clinically active disease (Table 3). An exploratory agreement analysis demonstrated Cohen’s kappa coefficient of 0.77, indicating substantial agreement between 18F-FDG PET/CT findings and clinical outcomes (Table 2).

In a subgroup analysis stratified by clinical indication, the diagnostic performance of 18F-FDG PET/CT differed slightly between initial staging and restaging examinations. For initial staging (*n* = 17), sensitivity was 75.0% (95% CI: 30.1–95.4%), specificity 92.3% (95% CI: 66.7–98.6%), PPV 75.0% (95% CI: 30.1–95.4%), NPV 92.3% (95% CI: 66.7–98.6%), and accuracy 88.2% (95% CI: 65.7–96.7%). For restaging (*n* = 14), sensitivity was 83.3% (95% CI: 43.6–97.0%), specificity 100% (95% CI: 67.6–100%), PPV 100% (95% CI: 56.6–100%), NPV 88.9% (95% CI: 56.5–98.0%), and accuracy 92.9% (95% CI: 68.5–98.7%). Given the small subgroup sizes and overlapping confidence intervals, these indication-based differences should be interpreted as descriptive rather than statistically significant (Table 4). These confidence intervals do not account for within-patient correlation and should be interpreted accordingly. In the restaging subgroup, eight of the nine PET-negative examinations were classified as true negative on follow-up, and one was subsequently confirmed as false-negative.

A subgroup analysis excluding all five spitzoid melanoma examinations (four TN and one TP) yielded sensitivity 77.8% (95% CI: 45.3–93.7%), specificity 94.1% (95% CI: 73.0–99.0%), PPV 87.5% (95% CI: 52.9–97.8%), NPV 88.9% (95% CI: 67.2–96.9%), and overall accuracy 88.5% in the remaining 26 examinations. These estimates were consistent with the full-cohort values, with no metric showing a substantial change upon exclusion of the spitzoid subgroup.

To assess the potential impact of incorporation bias arising from the post hoc adjudication of four initially suspicious examinations, sensitivity analyses were performed using two alternative classifications of the initial PET/CT interpretations (Supplementary Table S1). Of the four suspicious examinations, three were associated with active disease according to the reference standard (one subsequently adjudicated as true positive and two as false negative), while one was associated with disease absence (adjudicated as true negative). Treating all suspicious examinations as negative yielded a sensitivity of 70.0% (95% CI: 39.7–89.2%), specificity of 95.2% (95% CI: 77.3–99.2%), PPV of 87.5% (95% CI: 52.9–97.8%), and NPV of 87.0% (95% CI: 67.9–95.5%). Treating all suspicious examinations as positive yielded a sensitivity of 100% (95% CI: 72.2–100%), specificity of 90.5% (95% CI: 71.1–97.3%), PPV of 83.3% (95% CI: 55.2–95.3%), and NPV of 100% (95% CI: 83.2–100%). The adjudicated estimates (sensitivity 80.0%, specificity 95.2%) fell within the range defined by these two scenarios. Because adjudication incorporated information from the reference standard, the diagnostic performance estimates should be interpreted with consideration of potential incorporation bias.

## 4. Discussion

Malignant melanoma, although rare in the pediatric population, may exhibit aggressive clinical behavior and early dissemination [1], underscoring the importance of accurate staging and follow-up imaging [9,10,11,12]. In this retrospective cohort, we evaluated the clinical utility of 18F-FDG PET/CT in children with malignant melanoma and found that PET/CT provides clinically meaningful information, particularly during staging and restaging.

In accordance with current international guidelines [18,19], PET/CT is recommended for staging in Stage IIB and above. In our cohort, 14 patients (70%) had Stage II or III disease; however, because substage information was not available for the five patients with Stage II disease, the exact proportion meeting the Stage IIB criterion could not be determined. While sentinel lymph node biopsy (SLNB) remains the standard method for detecting microscopic nodal disease in early-stage melanoma, PET/CT serves a complementary role in evaluating macroscopic nodal involvement and distant metastases in higher-stage disease. Our findings are consistent with the potential complementary role of 18F-FDG PET/CT in guideline-based staging algorithms for pediatric melanoma, although dedicated evidence in this population remains limited.

A key finding of our study is the high negative predictive value of 18F-FDG PET/CT (90.9%) observed in this cohort. However, NPV depends on disease prevalence and patient selection, and two false-negative examinations were recorded; this finding should therefore be regarded as preliminary. In pediatric patients undergoing restaging due to clinical concern for recurrence, eight of the nine negative PET/CT examinations were associated with absence of clinically active disease during the available follow-up, which may support continued surveillance in conjunction with clinical assessment [20]. This potential rule-out utility, if confirmed in larger cohorts, would be especially valuable in pediatric oncology, where avoiding unnecessary invasive procedures and additional imaging is a major clinical priority. The predominance of adolescent patients in our cohort is consistent with previous reports indicating that melanoma is more frequently diagnosed during adolescence than early childhood [3].

In the subgroup analysis, restaging examinations showed particularly high specificity and PPV, suggesting that a positive 18F-FDG PET/CT finding during restaging is likely to reflect true recurrent or metastatic disease. However, given the wide and overlapping confidence intervals across subgroups, these indication-based differences should be considered preliminary, and larger studies are needed to determine whether diagnostic performance meaningfully differs between staging and restaging in pediatric melanoma.

Although pediatric melanoma differs from adult melanoma in incidence, biological behavior, and patterns of spread, the diagnostic performance observed in our cohort was comparable to that reported in adult populations [2]. In adult melanoma, a recent meta-analysis reported pooled sensitivity and specificity of 18F-FDG PET/CT of approximately 90% for detecting systemic metastases, with excellent overall diagnostic accuracy [10,11]. Although derived from adult populations, these findings provide an important reference point and are broadly consistent with the diagnostic performance observed in our pediatric cohort. These observations support the feasibility of 18F-FDG PET/CT in pediatric melanoma, particularly in the evaluation of suspected recurrence and advanced disease.

Of note, spitzoid melanoma represented a distinct histological subset in our cohort (*n* = 5, 25%), a proportion higher than typically reported in adult series. Spitzoid melanoma is biologically distinct from conventional melanoma, characterized by a superficial spreading growth pattern and a lower propensity for distant metastasis [21]. In line with this reported behavior, all spitzoid cases in our cohort demonstrated negative or true-positive PET/CT findings at initial staging, with no false-positive or false-negative results recorded in this subgroup. While the limited FDG avidity of spitzoid melanoma may reduce the sensitivity of PET/CT for primary tumor detection, whole-body imaging retains clinical value in this population by providing a comprehensive assessment for occult nodal and distant metastatic disease, particularly in higher-risk cases. Notably, exclusion of spitzoid examinations from the analysis did not meaningfully alter overall diagnostic performance metrics, suggesting that the performance estimates of 18F-FDG PET/CT in this cohort were not substantially influenced by the favorable biological profile of this subgroup.

Suspicious PET/CT findings in our series were confined to lymph nodes and reflected a cautious interpretative approach. Given the known influence of inflammatory and reactive processes on 18F-FDG uptake—especially in pediatric patients—such findings underscore the importance of clinical correlation, follow-up imaging, and histopathological confirmation when appropriate [22]. In this context, the negative predictive value observed in our study is encouraging but should be interpreted with caution. Notably, both false-negative examinations in our cohort were initially classified as suspicious and subsequently adjudicated as negative, despite evidence of active disease established through the reference standard. These findings underscore the importance of continued clinical surveillance and further diagnostic evaluation when PET/CT findings are equivocal.

While hybrid modalities such as PET/MRI may offer potential advantages in radiation dose reduction, their availability remains limited in routine clinical practice. In contrast, 18F-FDG PET/CT is widely accessible and represents the most established whole-body imaging modality in oncologic care. Additionally, advances in PET/CT technology, including low-dose CT protocols and optimized acquisition techniques, have enabled meaningful reductions in radiation dose. These factors make PET/CT a practical imaging modality for evaluating pediatric melanoma.

This study has some limitations. First, the retrospective design, small sample size, and the absence of systematic histopathological confirmation in PET-negative cases represent key limitations of this study. Second, due to the retrospective nature of the study, certain histopathological details, such as Breslow thickness and Clark level, were not available for all patients, as these patients were diagnosed and initially treated at different institutions or during earlier years when reporting practices were less standardized; consequently, these parameters could only be reported for a subset of the cohort. Third, image re-evaluation was performed with knowledge of clinical outcomes, potentially introducing interpretation bias. In particular, the adjudication of the four initially suspicious examinations incorporated information from the reference standard, which may have influenced the diagnostic performance estimates. To address this limitation, we also performed sensitivity analyses based on the initial PET/CT interpretations, treating suspicious findings as either positive or negative. Fourth, PET/CT examinations were acquired on two different scanner systems (Siemens Biograph TruePoint and GE Discovery IQ) with different reconstruction algorithms, potentially introducing variability in lesion detectability and SUV measurements. Fifth, retrospective case identification relied on a C43 diagnostic code search, and missed cases due to incomplete coding cannot be entirely excluded. Accordingly, the diagnostic performance metrics should be interpreted as exploratory. Sixth, as some patients contributed more than one PET/CT examination, the reported examination-level confidence intervals do not account for within-patient correlation; this limitation applies to all diagnostic performance metrics reported in this study. Nevertheless, given the rarity of pediatric melanoma and the limited availability of prospective data, our findings provide meaningful insight into the clinical utility of 18F-FDG PET/CT in this unique patient population. Larger, multicenter studies are warranted to validate these results and to help define standardized imaging strategies for pediatric melanoma staging and surveillance.

## 5. Conclusions

In this retrospective single-center study, 18F-FDG PET/CT demonstrated promising diagnostic performance for both staging and restaging of pediatric malignant melanoma, with high observed specificity and negative predictive value in this cohort. Given the small sample size, retrospective design, inclusion of multiple examinations from individual patients, and the absence of systematic histopathological confirmation in PET-negative cases, these findings should be interpreted as exploratory. Nevertheless, the results suggest that 18F-FDG PET/CT may contribute meaningfully to clinical decision-making in this rare pediatric malignancy, particularly within a multidisciplinary assessment framework.

## Figures and Tables

**Table 1:** Patient characteristics.

Characteristic	*n*	%
**Number of patients**	20	—
**Sex**		
Female	12	60
Male	8	40
**Age at imaging (years)**		
Mean ± SD (range)	11 ± 4 (4–18)	—
**Age group**		
Children (1–11 years)	9	45
Adolescents (12–18 years)	11	55
**Mean follow-up duration**	97.8 months	—
**Primary tumor location**		
Lower extremity	7	35
Upper extremity	5	25
Trunk	5	25
Head–neck	3	15
**Melanoma subtype**		
Conventional melanoma	9	45
Spitzoid melanoma	5	25
Melanoma in congenital melanocytic nevus	5	25
—Trunk	3	—
—Extremity	2	—
Conjunctival melanoma	1	5
**Clinical stage**		
Stage I	6	30
Stage II	5	25
Stage III	9	45
**Breslow thickness**		
Mean (mm)	5.5 (*n*: 11)	
**Clark level**		
Mean level	IV (*n*: 8)	

**Table 2:** Summary of 18F-FDG PET/CT findings.

Parameter	Value
**Total PET/CT examinations**	31
**Indication**	
Initial staging	17 (54.8%)
Restaging	14 (45.2%)
**Examination-Based PET/CT Interpretation**	
Negative	22 (71.0%)
Positive	9 (29.0%)
Equivocal	0 *
**Patterns of PET-Positive Disease (Examination-Based)**	
Local recurrence	1
Isolated lymph node involvement	5 †
Combined local and nodal disease	2
Distant metastatic disease	1
Patient-Based PET/CT Outcome **	
Consistently PET-negative	12 (60%)
At least one PET-positive scan	6 (30%)
False-negative result	2 (10%)
False-positive result	1 (5%)
**Diagnostic Performance (Examination-Based)**	
Sensitivity	80.0% (95% CI: 49.0–94.3%)
Specificity	95.2% (95% CI: 77.3–99.2%)
Positive predictive value (PPV)	88.9% (95% CI: 56.5–98.0%)
Negative predictive value (NPV)	90.9% (95% CI: 72.2–97.5%)
**Overall accuracy**	90.3% (95% CI: 75.1–96.7%)
**Agreement Analysis**	
**Cohen’s κ**	0.77 (95% CI: 0.47–1.00)

* Four examinations were initially classified as suspicious. Following histopathological confirmation or clinical and imaging follow-up, one was adjudicated as positive (true positive) and three as negative (one true negative and two false negatives). No examination remained equivocal in the final analysis. The single false-positive examination was initially interpreted as positive. Estimates based on the initial interpretations are provided in Supplementary Table S1. ** One patient had both a false-negative and a subsequent true-positive examination during follow-up; patient-based categories are not mutually exclusive. † One examination in this category represented a false-positive finding that proved clinically negative on follow-up.

**Table 3:** Cross-tabulation of 18F-FDG PET/CT findings versus reference standard (examination-based, *n* = 31).

18F-FDG PET/CT Result	Clinical Reference (+)	Clinical Reference (–)	Total
**PET-positive**	8 (TP)	1 (FP)	9
**PET-negative**	2 (FN)	20 (TN)	22
**Total**	10	21	31

TP, true positive; FP, false positive; FN, false negative; TN, true negative. Sensitivity 80.0%, specificity 95.2%, PPV 88.9%, NPV 90.9%, overall accuracy 90.3% (see Table 2 for 95% confidence intervals). For estimates based on the initial interpretations, see Supplementary Table S1.

**Table 4:** Subgroup diagnostic performance of 18F-FDG PET/CT by clinical indication.

Parameter	Initial Staging (*n* = 17)	Restaging (*n* = 14)
**Raw counts**		
TP	3	5
FP	1	0
FN	1	1
TN	12	8
**Diagnostic performance**		
Sensitivity	75.0% (95% CI: 30.1–95.4%)	83.3% (95% CI: 43.6–97.0%)
Specificity	92.3% (95% CI: 66.7–98.6%)	100% (95% CI: 67.6–100%)
PPV	75.0% (95% CI: 30.1–95.4%)	100% (95% CI: 56.6–100%)
NPV	92.3% (95% CI: 66.7–98.6%)	88.9% (95% CI: 56.5–98.0%)
Overall accuracy	88.2% (95% CI: 65.7–96.7%)	92.9% (95% CI: 68.5–98.7%)

CI, confidence interval; TP, true positive; FP, false positive; FN, false negative; TN, true negative; PPV, positive predictive value; NPV, negative predictive value. Confidence intervals were calculated using the Wilson score method and do not account for within-patient correlation arising from repeated examinations in individual patients.

## Data Availability

The data presented in this study are available upon request from the corresponding author, due to institutional and patient privacy restrictions.

## Supplementary Materials

The following supporting information can be downloaded at https://www.mdpi.com/article/10.3390/children13101428/s1, Table S1: 3×2 contingency table of initial 18F-FDG PET/CT interpretation against reference standard outcome. Four examinations were initially classified as suspicious; three were associated with active disease according to the reference standard (one adjudicated as positive → true positive; two adjudicated as negative → false negative) and one with disease absence (adjudicated as negative → true negative). Treating all suspicious examinations as negative: sensitivity 70.0% (95% CI: 39.7–89.2%), specificity 95.2% (95% CI: 77.3–99.2%). Treating all suspicious examinations as positive: sensitivity 100% (95% CI: 72.2–100%), specificity 90.5% (95% CI: 71.1–97.3%). Adjudicated classification: sensitivity 80.0%, specificity 95.2% (Table 3).
