# Supplementary material for: Clinical Utility of 18F-FDG PET/CT for Staging and Restaging of Pediatric Malignant Melanoma: A Single-Center Retrospective Study
Source: Children (Basel). 2026 Oct 18;13(10):1428. doi: 10.3390/children13101428 (PMC13703103; doi:10.3390/children13101428)
Supplement: Supplementary file 1 [file children-13-01428-s001.zip › children-4586672-supplementary.pdf]

Supplementary Table S1: 3×2 contingency table of initial 18F-FDG PET/CT interpretation against reference standard outcome. Four examinations were initially classified as suspicious; three were associated with active disease according to the reference standard (one adjudicated as positive → true positive; two adjudicated as negative → false negative) and one with disease absence (adjudicated as negative → true negative). Treating all suspicious examinations as negative: sensitivity 70.0% (95% CI: 39.7–89.2%), specificity 95.2% (95% CI: 77.3–99.2%). Treating all suspicious examinations as positive: sensitivity 100% (95% CI: 72.2–100%), specificity 90.5% (95% CI: 71.1–97.3%). Adjudicated classification: sensitivity 80.0%, specificity 95.2% (Table 3).

Initial PET/CT Interpretation	Reference Standard: Disease Present (n=10)	Reference Standard: Disease Absent (n=21)
**Positive**	7	1
**Suspicious**	3	1
**Negative**	0	19
**Total**	**10**	**21**
